# Governance of Assisted Living in Long-Term Care: A Systematic Literature Review

**DOI:** 10.3390/ijerph182111352

**Published:** 2021-10-28

**Authors:** Luting Poh, Si-Ying Tan, Jeremy Lim

**Affiliations:** 1 Memory Aging and Cognition Centre, Department of Pharmacology, Yong Loo Lin School of Medicine, National University of Singapore, 10 Medical Dr., Singapore 119260, Singapore; e0292624@u.nus.edu; 2 Leadership Institute for Global Health Transformation, Saw Swee Hock School of Public Health, National University of Singapore, 21 Lower Kent Ridge Rd., Singapore 119260, Singapore

**Keywords:** assisted living, long-term care, ageing, governance, regulation, policy, review

## Abstract

Assisted living (AL) is an emerging model of care in countries where long-term care needs are escalating, with emphasis given to promoting independence and autonomy among the residents to achieve active and healthy ageing. Unlike established nursing homes, the governance of AL is nebulous due to its novelty and diverse nature of operations in many jurisdictions. A comprehensive understanding of how AL is governed globally is important to inform regulatory policies as the adoption of AL increases. A systematic literature review was undertaken to understand the different levels of regulations that need to be instituted to govern AL effectively. A total of 65 studies, conducted between 1990 to 2020, identified from three major databases (PubMed, Medline, and Scopus), were included. Using a thematic synthesis analytical approach, we identified macro-level regulations (operational authorisation, care quality assessment and infrastructural requirements), meso-level regulations (operational management, staff management and distribution, service provision and care monitoring, and crisis management), and micro-level regulations (clear criteria for resident admission and staff hiring) that are important in the governance of AL. Large-scale adoption of AL without compromising the quality, equity and affordability would require clear provisions of micro-, meso- and macro-level regulations.

## 1. Introduction

### 1.1. Definitions and Operationalisations of Assisted Living (AL) in the Literature

The development of assisted living (AL) took root in North America around the late 1970s, a time when there was a growing consumers’ dissatisfaction towards how nursing homes were organised and managed in the US due to high rates of exposed abuse cases, which resulted in low public trust [1]. In the next two decades, assisted living facilities (ALFs) expanded rapidly in the US long-term care market, ushered in by a paradigm shift from conventional nursing homes to residential care facilities that was driven by a consumer-centred care philosophy [1,2]. According to the National Centre for Assisted Living (NCAL) in the US, assisted living grounds on the philosophy of providing person-centred care to meet the residents’ specific needs and preferences, and aim to serve individuals who need help in activities of daily living and health services but do not require round the clock nursing services for the extended duration [3]. The primary purpose of an ALF is to promote better physical, mental and psychological health of individuals who seek to maintain independence and autonomy even when they require a certain degree of assistance in daily living [4]. Additionally, AL settings were reported to endow the residents with more privacy and individual freedom as opposed to nursing homes [5]. By preserving their overall functionality, AL enables older residents to enjoy healthy and active ageing [6].

Even though the concepts and original intent of AL are alluring, in reality, the implementation of AL is, however, heterogeneous and convoluted. This is due to the variability in implementation structures, processes, resident profiles, and philosophies of the facilities in different jurisdictions [7]. In the US, Brown Wilson came up with a typology to classify different service approaches to AL that existed in the mid-1990s to 1990s—hybrid, hospitality, housing and healthcare [1]. Quite similarly, Stone classified AL provider models into independent housing with services, freestanding private residential AL, nursing home expansion, continuing care retirement communities, and comprehensive health and long-term care services which were designed as part of a suite of services in the integrated acute care system offered in the hospital [8].

### 1.2. AL Versus Nursing Home Care

Various characteristics differentiate AL from other conventional care models, such as nursing homes. First, AL offers its residents a greater degree of independence than those staying in nursing homes. The level of care and monitoring which AL residents require are generally not as intensive as nursing home residents [5]. Second, ALFs provide medical care and services upon the request of the residents, allowing the residents to exercise higher levels of autonomy in making decisions related to their care [9]. Scheduled care like this is not a common feature of nursing homes in which service provisions tend to be more pre-determined and directive. Overall, the goal of AL is to effectively facilitate healthy and active ageing while taking care of those with medical needs.

### 1.3. The Global Phenomenon of Ageing Population and the Need for Service Diversification in Long-Term Care

The world’s population has been rapidly ageing in the past three decades. In 1990, there were 703 million people aged 65 and above; by 2019, this number had doubled. This translates to an increment from 6% to 9% in the share of the population aged 65 and above in three decades. Likewise, the eldest old population (aged 80 and above) tripled between 1990 and 2019, with the effects most significantly observed in Asia and North Africa. Correspondingly, life expectancy also increased substantially in line with socio-economic improvement in the past few decades [10]. This ageing phenomenon will accelerate the demand for care in many ageing countries. Hence, governments will have to respond to this escalating need in long-term care through service diversification and expansion [11,12,13].

Despite having roots in North America, AL is emerging as a mainstream model and philosophy of care for older people and is increasingly being adopted in ageing Asian countries [14,15,16,17]. With the changing demographic structures in many industrialised countries whereby the need for residential care services has increased dramatically, AL will emerge as an increasingly popular long-term care model due to its privacy- and autonomy- preserving characteristics [18].

### 1.4. Knowledge Gaps and Aim of the Review

The governance of AL remains less explicit than the other care options in the long-term care spectrum as its implementation and regulatory enforcement remain understudied to date. Currently, the market for AL is not heavily regulated by governments across the world [19]. Even though the lack of standardisation of regulations allows different ALFs to have more flexibility and adapt their operational and resident strategies to meet the market demands [20], this could expose the AL residents to possible financial exploitation by the service provider in a free market setting, as observed in the US and Israel during the contract renewal processes that often involved complex fee structures [21,22,23]. Therefore, explicit regulatory provisions from the government are needed to enable the government to balance both consumers’ and the providers’ interests, and to protect the interests of the older adults and low-wage workers in the ALFs.

Some recent studies have reviewed and examined the regulatory approaches of AL, focusing specifically on facilities for residents with dementia [4,23], as well as key aspects of quality of life (QoL) in ALFs [7,24,25,26,27,28,29]. While these studies contribute to the understanding of AL, they possess several limitations. The above studies generally focused only on one or two aspects of QoL in ALFs, such as the services provided [26], staffing requirements [7], admission and training [27]. For studies that covered more areas, the investigations and discussion mainly revolved around the regulations for residents with dementia [4,23]. Similarly, two other studies also provided a focused review on the state’s regulation for ALFs during the outbreak of infectious diseases, including the most recent Covid-19 pandemic [27,28]. None of these studies have addressed the issue of governance. By and large, the current state of evidence provides a rather compartmentalised understanding of how AL is governed as a long-term care model. Overall, there is a lack of clarity on the entire *modus operandi* of AL in different parts of the world, especially regarding issues around their implementation structures and governance frameworks. To address these knowledge gaps, this review aims to examine the regulations of AL using the lens of governance. This would entail understanding various aspects of the AL regulations, including but not limited to institutional structures, operational processes, quality assurance and assessment across the world. To achieve the above aim, we pose the following review question: What are the different levels of regulations that need to be in place to govern AL effectively?

## 2. Conceptual Framework

In this review, we applied a three-level governance framework that specifies macro-, meso-, and micro-level governance strategies in synthesising and consolidating our results [30,31]. In this review, we adopted the conceptual framework from the original framework developed by Roberts (2019) by using a combination of deductive and inductive approaches.

At the macro-level, overarching strategies were pursued to achieve sectoral wide priorities which defined the foundation and architecture of the state or industry [30]. The macro-level regulation refers to the implementation of policies across the sector to direct the market’s growth and to safeguard the interest of the consumer. The actors working at this level entailed state policymakers, advocacy coalitions, agencies and different interest groups [30,31,32].

At the meso-level, governance strategies were used to shape operational guidelines and structures of the organisations or institutions [30]. The meso-level regulation occurred when the authority imposed rules and guidelines to the service providers to construct their own operational frameworks based on the state’s requirements [33].

At the micro-level, governance strategies entailed examining the behaviours and preferences of the actors within the institutional apparatuses who were subjected to their authorities [30]. Regulations at the micro-level targeted individuals involved in a particular setting [31,33] who in the context of this review referred mainly to the consumers and the ALF staff.

The conceptual approach of dissecting the governance of AL into macro, meso and micro levels helped to address different levels of concerns, as well as to categorise the important actors at each level. This approach would also enable specific illustrations on how governance strategies for AL can be applied and exercised at all levels of regulation.

## 3. Methods

### 3.1. Search Strategy

We undertook a systematic literature review to systematically identify, analyse and synthesise literature that capture policy and governance processes regarding AL across the world. We included academic sources from multiple disciplines, such as medical, public health, policy sciences, and social sciences journals. Three academic databases that are the most widely used repositories in public health and social sciences (PubMed, Medline and Scopus) were utilised as data sources in the data-gathering process. We performed the searches from January to February 2021. Our search string captures both ‘*assisted living*’ and the regulatory aspects of assisted living which this review intends to examine including *‘governance’, ‘regulation’, ‘law’ and ‘legislation’*. We utilised wildcard, truncation and hyphen characters to maximise the evidence search.

### 3.2. Inclusion and Exclusion Criteria

Three inclusion criteria were applied to facilitate study selection in the study screening process. First, we included only literature published in the English language. Second, we included three decades of literature (both academic and grey literature) that were published between 1990 to February 2021. The starting year (1990) was decided based on the fact that AL first took root in the west, especially, North America, beginning in the 1990s [34,35]. Third, we included both conceptual and empirical studies (quantitative and qualitative) examining public policy, governance and regulations of AL which explicitly include these specific components and dimensions: (i) definitions and conceptualisations, (ii) background and implementation of AL, such as resident profiles, institutional structure, models of delivery and operational design, (iii) regulatory aspect focuses on quality assurance (admission systems, staff management and distribution, training, care monitoring and service provision, responses to the Covid-19 pandemic, quality assessment), and, (iv) modes of regulation.

We also applied four exclusion criteria to filter studies that are irrelevant and fall out of the scope of this review. First, we excluded studies that were not published in the English language. Second, we excluded studies that were published before the year 1990. Third, we excluded social work, sociology, anthropology and sociology studies that examined lived experiences residents’ or providers’ perceptions of AL from the residents’ or providers’ perspectives. Fourth, we excluded historical analyses of long-term care policies and AL development in a country.

### 3.3. Data Extraction

A data extraction framework was constructed by the first and second authors. This framework included information, such as the title of the study, the countries in which the study was conducted, the aims/objectives of the study, the methods/design of the study, and the three levels of regulations examined in this review. The information was extracted and consolidated in a spreadsheet by using a data table to aid the data analysis process. All data were cross-checked for consistency and validity.

### 3.4. Critical Appraisal of Included Studies

We adopted Crowe’s critical appraisal tool (CCAT) to critically appraise the quality of each included study. The CCAT tool is relevant due to the heterogeneity of study designs in this review as it enables a wide range of research designs (quantitative, qualitative, and mixed-method studies) to be evaluated. Additionally, it also offers a high degree of reliability [36,37]. The CCAT has eight category items (Preliminaries, Introduction, Design, Sampling, Data Collection, Ethical Matters, Results and Discussion), with each category item scoring five points and a total aggregate score of 40. The CCAT User Guide offered detailed descriptions and references on how each category item can be scored [38]. (See Supplementary Materials for details). The first author applied CCAT to all the studies, and the second author did the same for more than 50% of the studies. This process was conducted independently, and discrepancies were resolved through ongoing discussion.

### 3.5. Synthesis and Analysis of Results

**Figure 1:**
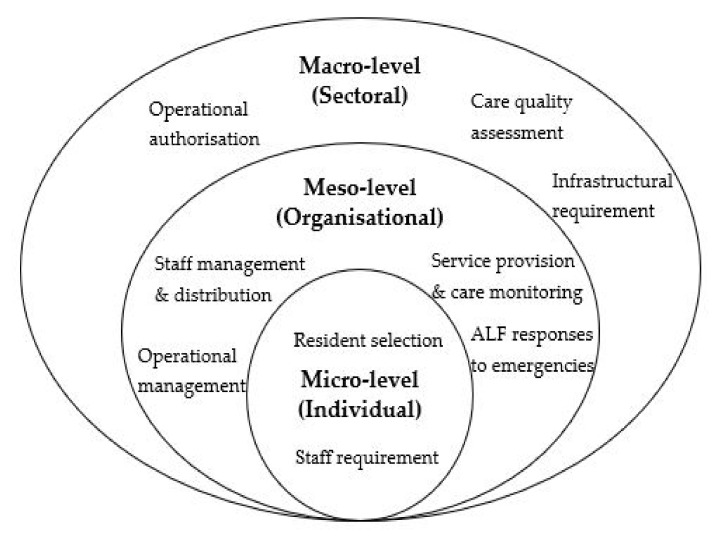
Governance framework for AL.

Using a combination of deductive and inductive approaches, we first anchored on the definitions of the micro-, meso-, and macro-levels governance strategies coined by Roberts (2019) and used these to classify the regulatory strategies of AL deductively from the initial scoping of the relevant studies. Inductive approaches were later followed to develop precise themes in each regulatory level, accounting for the unique actors, institutions and processes involved in each regulatory level. Strategies observed at the macro-level commonly involved operational authorisation, quality assessment and infrastructural requirements [31]. At the meso-level, staff management and distribution, service provision and care monitoring, operational management and emergency response were postulated to be important strategies in the regulation of AL [39]. At the micro level, through the facilities’ admission systems, the state legislature regulated the resident selection and matched their needs profiles with the facilities’ capacities [39]. Similarly, ALFs were required to hire their staff according to the state’s requirements [3]. Figure 1 illustrates the conceptual framework that was modified from the initial framework by Roberts (2019).

Thematic synthesis was employed as the data analysis strategy in this review [40]. Thematic synthesis is one of the most applied narrative synthesis approach often utilised to synthesise evidence that is primarily qualitative in nature and used to inform policy and practice [41,42]. As most studies included in the review are qualitative empirical studies or conceptual studies, thematic synthesis enables us to streamline the data analysis process in a three-stage process. This three-stage process entails line-by-line coding, formulation of descriptive themes and development of analytical themes. Line-by-line coding allows us to read through the extracted data carefully to understand their explicit and underlying meanings. This process paved the way for the development of descriptive themes that cover the three levels of governance (micro, meso, and macro) as demonstrated in the conceptual framework. Analytical themes were formed by merging descriptive themes that are identical and drawing connections to those that appear to be somewhat similar to formulate distinct themes that mimic the different regulatory dimensions in the conceptual framework of AL governance [40].

## 4. Results

### 4.1. Contexts and Characteristics of the Studies

**Figure 2:**
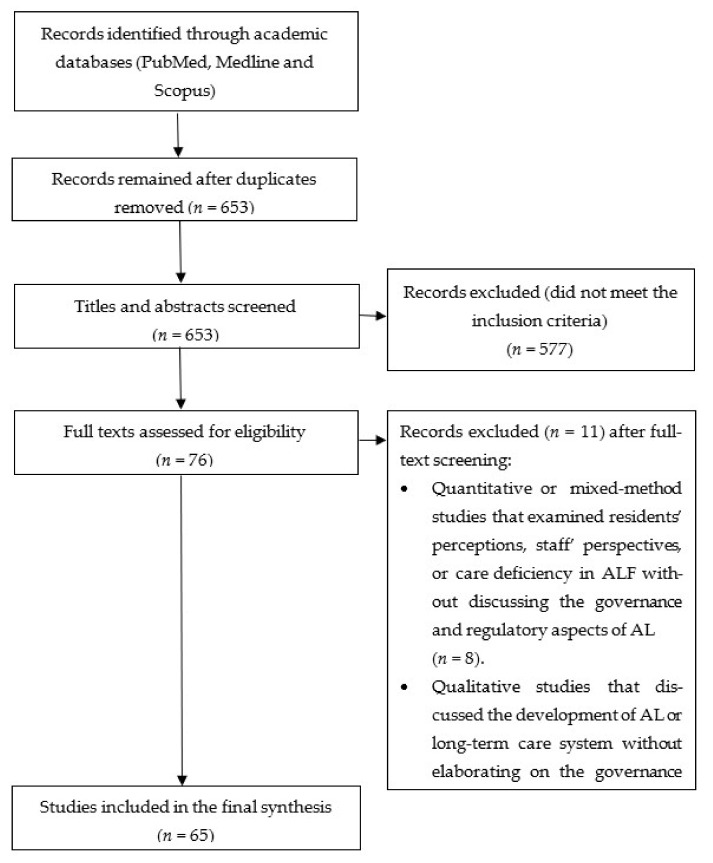
PRISMA flow diagram of the literature search, selection process and reasons for exclusion.

A total of 65 studies that met the inclusion criteria were included in the final synthesis. Figure 2 reported the Preferred Reporting Items for Systematic Reviews and Meta-Analyses (PRISMA) flowchart exhibiting the details of evidence search processes at various stages.

The 65 studies included in this systemic literature review encapsulated the essential elements of AL as a type of long-term care facility and presented the respective regulatory approaches in the United States, Canada and Israel. Amongst them, most of the studies were conducted in the US [2,4,6,7,8,19,20,21,23,24,25,27,28,29,34,35,43,44,45,46,47,48,49,50,51,52,53,54,55,56,57,58,59,60,61,62,63,64,65,66,67,68,69,70,71,72,73,74,75,76,77,78,79,80,81,82,83,84,85,86,87]. There were also two studies from Canada [26,88], and one from Israel [22]. In terms of the study design, 31 studies were qualitative conceptual studies which reviewed the different aspects of AL models and examined their potential challenges [4,6,7,8,19,20,22,23,24,27,28,29,34,35,43,44,45,46,47,48,49,50,51,52,53,54,55,56,57,85,86]; 19 studies were based on surveys conducted with the AL service providers, nursing staffs, residents and their family members [21,25,26,58,59,60,61,62,63,64,65,66,67,68,69,70,71,87,88]; three studies were observational studies conducted in the US, investigating state variations in AL regulatory policies, conditions of people living with dementia in the AL settings and rates of medication errors committed by AL staff [65,72,73]; five studies performed analysis on state-level official databases to analyse the institutional arrangements of AL focusing specifically on facility staffing, training, inspection, and enforcement regulations in the US [2,74,75,76,77]; three literature reviews discussed the definitions and classifications of AL [78,79,86] and two retrospective cohort studies focused on AL financing and services [80,81]. The remaining three studies were a systematic analysis, an ethnographic study, and a cross-sectional descriptive study addressing the issues related to infectious disease control, medication management and medical care delivery [82,83,84] (see Appendix A for details of the studies).

### 4.2. Critical Appraisal of the Sources of Evidence

Based on the scoring system of CCAT, forty-one studies were considered high-quality evidence as they scored more than 30 points. Generally, these studies were given at least 4 points in the following categories: preliminaries, introduction, design, sampling, data collection, results and discussion. The majority were survey studies that provided clear descriptions of their aims and objectives, data collection methods and result analysis. Another ten studies were considered as evidence with moderate quality as they scored between 20 to 29 points. The remaining fourteen studies were of lower quality and scored less than 20 points mainly due to missing or lack of information in describing/addressing study designs, sampling frames, data collection methods, and ethical matters.

### 4.3. Macro-Level Regulation

#### 4.3.1. ALF Operational Authorisation

There are primarily four modes of regulation in the governance of AL across the North American region—licensure or certification, agency review or on-site inspection, applying market-based tool in regulation, and regulatory inaction [4,6,19,20,22,25,26,27,45,48,51,60,62,71,73,77].

Licensure or certification was the most common mode of regulation of ALFs. It is usually conducted by setting minimum standards of care for ALFs, encompassing areas, such as admission requirements, housing and accommodation, frequency and extent of care services which included medication administration, building and facility requirements, residents capacity, payment policies, staffing levels and experiences, environment safety, as well as infection prevention and control [4,6,19,20,22,25,26,27,48,51,60,62,71]. In terms of licensing regime, some studies proposed using a classification system [19,22], or setting up different licensure categories [25], to delineate the different types of responsibilities and liabilities involved for different types of ALFs. For example, in Texas, a multi-tiered classification system was established in the licensure of ALFs. ALFs can apply to be licensed under “Type A”, “Type B”, or “Type C” facilities depending on the care requirements and types of residents that they plan to admit [19]. Likewise, in Florida, ALFs licensure was divided into either facilities with “limited nursing services” which provided standard services, “extended congregated care” that offered extra care services beyond standard and routine care, or “limited mental health” that provided specialised behavioural care programs for residents who were diagnosed with several mental illnesses [25].

Apart from licensure or certification, agency review was another regulatory approach proposed as an enforcement or correction plan for non-compliant ALFs [4,73,77]. In addition to routine inspection as part of the licensure requirements, ALFs would also be subjected to on-site inspection whenever a complaint was filed. Typically, an independent ALF surveyor could be engaged for this purpose [77]. In the event of any breaches of duty, different types of penalties or fines would be imposed. These included immediate suspension of the facility by revoking facility licenses, denying licenses renewal, limiting new admissions or service provisions, regular monitoring from the authorities, mandatory transferring of residents to other facilities and fines levied [77].

One study had proposed the use of a market-based tool, such as pay-for-performance in the regulation of ALFs [62]. Using a selection of quality indicators or performance measures to reward providers with monetary reward. Pay-for-performance was touted as a payment incentive that was effective to promote quality improvement among the ALFs by stimulating the investment in internal quality improvement by the providers. Nevertheless, there were potential drawbacks to this incentive-based system as it could potentially create complicated incentives that may not result in overall quality improvement. Depending on how the incentives were designed, pay-for-performance may at times propel providers to game the system through targeted improvement of several areas of care instead of focusing on holistic improvement of the entire care process [62].

Regulatory inaction was another possible approach to service authorisation [4,45]. Some of the possible reasons for regulatory inaction included the lack of information and understanding of the governance of ALFs, limited resources to enforce regulations, lack of consistency in policy direction and leadership, resistance from the industry and lack of public demand [4]. Regulatory inaction was deemed an undesirable approach which could result in suboptimal outcomes, such as public ignorance and provider ambiguities regarding the scopes and quality of care in ALFs [4]. It can also lead to abuse of residents, their families, and AL staff.

#### 4.3.2. Care Quality Assessment

Many factors influenced the quality of care offered by the ALFs. To ensure that the residents receive appropriate care during their stay in the ALF, it is essential for the state to set guidelines for the facility to adhere to. Facility owners need to constantly review and modify their policies and operations according to the state’s regulations to meet the scheduled and unscheduled needs of the residents. Based on the AL philosophies, our findings highlighted several tenets that guided the quality indicators employed by most ALFs [43,89]. First, most states required ALFs to provide key services, such as personal care, health-related care and ad-hoc services. For example, an ALF had to provide 24-h round the clock staff, housekeeping, at least two meals a day, be able to help with at least two activities of daily living (ADL) and assist in medication administration. Next, ALFs need to ensure that the services were designed to maximise residents’ dignity, autonomy, independence and safety. This can be reflected in the emphasis on privacy and independence in the infrastructural design where the resident can choose to stay in a single unit with personalised furniture, storage space for personal possessions and public space for dining and socialising [43,54]. Finally, some states required a more direct examination of the effectiveness of the measures mentioned earlier. Other than focusing on the facility’s performance and residents’ satisfaction, the outcome indicators should also evaluate the residents’ views on the level of individual autonomy, interaction with facility staff and members, and their unmet needs. Moreover, duration of stay, discharge reason and subsequent care location would also provide a more comprehensive understanding of the quality of service offered in ALF [43,90]. Several states actively assessed ALF’s quality of care by developing and administrating instruments to gauge the residents’ and family members’ satisfaction. While most of the states in the US do not have resident satisfaction instruments at the moment, several states including Ohio, Oregon, Washington, Wisconsin and Minnesota, are actively making an effort to develop instruments that assessed ALF’s quality of care by gathering feedback from the residents and family members [91]. Cognitive screening was often used to determine the residents’ ability to participate in data collection; family members would be providing feedback if the resident cannot do so. It is important for states and governments to deploy satisfaction instruments, as studies had shown that higher satisfaction was associated with more services or amenities, privacy, and a cohesive environment; whilst inexperienced staff, high staff turnover rate and medication-related issues were the common problems identified [20,34,90].

#### 4.3.3. Infrastructural Requirements

In the US, it is clear that although the roles of ALFs vary from state to state, ALFs generally serve a healthier population as compared to nursing homes or medical facilities [3]. Proper facility design plays a vital role in creating a non-institutionalised care environment based on the AL philosophies. Depending on a state’s definition of AL, ALFs can be regulated via several ways: AL licensing schemes from the state agencies, the state healthcare financing schemes or the state-funded AL programmes, the “Assisted Living Facility and Service Contract” between state agencies and facilities which varied in different locations, as well as recommendations from authorised advisory committees. Moreover, ALFs ought to adhere to the local building and fire codes based on the nature of their constructions and occupancies [92,93].

Based on the description of Herd (2001), the physical structure of ALF varies considerably and some resemble an upscale apartment building with a hotel-like distribution of residential units [34]. According to Kissam et al. (2003), the design of the infrastructure followed three fundamental principles: (1) to provide privacy and home-like amenities in addition to public and social recreational space, (2) to provide maximum autonomy while accommodating to the residents’ choices of care, and (3) to provide non-medical services, such as meals, personal care, supervision and basic housekeeping [6]. States with a more mature AL market (i.e., Maryland, Massachusetts, New Jersey, Ohio, Oregon and Washington) had clearer specifications regarding their unit requirements. As such, many ALFs in these states offered private residential units that included attached bathrooms and limited cooking or food storage facilities, with common dining and living areas for their resident. While most residents were allowed to move freely around the facility, Denham (2018) reported that residents with dementia would have to stay in units with restricted access to prevent them from wandering around and getting lost [35]. According to Carder (2017) and Kaskie et al. (2015), 25 states required ALFs to have egress features that restricted residents from leaving the facility unescorted. For example, facilities can use double alarm systems and tab alarms to alert the staff when residents with dementia left their premises [4,23]. On top of that, ALFs were also required to provide safe and secure outdoor areas for the residents by building accessible walkways, elderly-friendly stairwells, conducive exercise corners and shelters [4]. Sharp, toxic and hazardous objects should be kept in a locked location that was not accessible by the residents [23]. Regulations aside, many ALFs encouraged their residents to decorate and furnish their rooms with their belongings to create a sense of belonging and a home-like environment [4,34,57]. Overall, the infrastructural design of an ALF is an essential component of ALF regulation and should be constantly reviewed as residents’ needs evolve.

### 4.4. Meso-Level Regulation

#### 4.4.1. Staff Management and Distribution

Most ALFs possessed unique compositions of staff and personnel different from nursing homes. As reported in two surveys conducted in the US, the staff population comprised 7–36% of registered nurses (RNs), 9–27% of licensed practical nurses (LPNs), 13% of medication technicians, 26% certified nursing assistants (CNAs), 19% personal care aide (PCA) and 25% of other staff with non-medical background (i.e., housekeeping, dining staff) [58,87]. The ALFs were expected to fulfil specific state regulations regarding staff distribution in the US. For example, most states required a sufficient number of staff with particular qualifications to meet a 24-h schedule (though they may not necessarily be required to be physically present in the facilities) [79] and in most cases, the hiring of licensed staff (RN/LPN) was a requirement [77]. Half of the states also expected their ALFs to have at least one licensed nurse (RN/LPN) as a permanent/contract staff in the facility. If an ALF had special care unit like a dementia unit, the minimal requirement is to have a psychologist/physician, one registered nurse (with a background in dementia care), or two dementia-trained staff members to be on duty at all times [23]. Certain states also required the presence of a social worker and an administrator with relevant training to provide better care for residents with dementia [4].

It was common for ALFs to employ RN/LPN as full-time or part-time staff. Most of the ALFs had a nurse (RN/LPN) present during the day. In comparison, some small facilities with a median capacity of 22 beds had no in-house but contract-based RN or LPN [4,87]. Under certain states’ guidelines, ALFs can assign RNs to provide training to all staff members and residents and conduct preadmission assessments (such as the ability to self-administer medication) for the residents [7,49,50]. They also delegated duties to LPN/LVNs responsible for the care planning and the supervision of residents with stable conditions [49]. Other than nurses, there were also CNAs and PCAs that took up the role of direct care workers; staff social workers who functioned as psychosocial resources, and physicians who made on-site visits [44,55,83]. Though corporate-run ALFs usually had a nursing or a medical director, it was not a requirement from the regulatory perspective [44,61,64]. In terms of the skill levels, most of the staff were able to implement post-hospitalisation treatment recommendations and monitor blood pressure. However, not all the staff were trained to provide support on mental health issues and handle acute medical problems experienced by the residents [83].

#### 4.4.2. Service Provision and Care Monitoring

AL is a care philosophy that aims to provide a home-like environment care support for elderly who are no longer able to live independently but do not require the high-intensity care provided in nursing homes [54]. ALFs usually offer basic personal care, assistance with daily living activities, social services, recreational services, and medication management [77]. The minimum requirements stipulated by the states included providing round-the-clock assistance, meals, housekeeping and laundry services to the resident. In terms of ADLs, ALF staff would also need to help the residents in dressing, bathing, walking and toileting. Additional social and recreational activities, such as exercise and big group dining could also be organised in the ALF [55,86].

In the US, services offered in ALFs can be regulated via facility assessment and licensure. Different states offered different types of ALF licensing schemes, depending on the services available, the presence of specialised units (i.e., dementia care unit and mental health care unit) and the nature of care services offered. Most in-house staff were allowed to perform basic services. With training, in-house staff could also offer technically complex services. Similarly, staff trained in assessment, wound care, and therapies could conduct depression and mental health assessments, wound dressing, aromatherapy, influenza vaccinations, urinary catheterisations and stool card testing. On the other hand, specialty services, such as X-rays, mental health therapy, physical therapy, massage therapy, and hospice were usually contracted or outsourced to third-party providers. Among the services provided in ALFs, gastrostomy and intravenous medication were considered as specialised services that 55% of ALF did not provide [87].

Several studies suggested various regulatory measures to improve the care services in ALFs. First, implementing state regulatory policies by accounting for consumer preferences would cultivate a more efficient care delivery model [43,82]. Next, providing specific guidelines on the shared/negotiated risk agreement between an ALF and the residents would help to balance the needs to preserve the residents’ autonomy and to comply to the ALF’s legal obligations [44]. Most importantly, standardised instrument or protocol (i.e., Mini-Mental Status Examination, Minimum Data Set, Medication Self-Administration Assessment, Medication Management Instrument for Deficiencies in the Elderly) should be included in the regulation to guide the development of care planning, and assess residents’ medication self-administration abilities, and establish treatment regulations [44,48,61].

In terms of care monitoring, it was also important to monitor the unlicensed staff who were tasked to administer medication. In some ALFs, clinical skills or performance observations were conducted by either a pharmacist or a RN to ensure that unlicensed staff followed the guidelines and possessed clinical competencies to provide medication assistance to the residents [50].

#### 4.4.3. Operational Management

Though ALFs are not perceived as medical institutions, they provide a diverse range of healthcare services. Therefore, additional considerations have to be incorporated into the operations of ALFs. The most common medical-related service that ALFs offer is medication administration. Many ALFs assigned medication technician staff or registered nurses to manage the residents’ medication needs [58,61]. Many ALFs obtained the residents’ medications via a primary pharmacy and dispensed them using blister packs or traditional bottles of multiple doses [61]. The ALFs need to design specific operational policies addressing medication storage, medication error reporting, medication disposal and accountability for controlled drugs [61,64]. In addition, ALFs had to take responsibility for managing the healthcare needs of the residents. ALFs could choose to manage the medical conditions of the residents within their premises as long as this was within their provision capacities. However, when the residents’ medical needs exceeded their capacities, they would have to direct the individuals to nursing homes or hospitals [8,20]. In most states in the US, ALFs were more inclined to retain most dependent residents for practical reasons, such as revenue retention. Additionally, relocation can induce unnecessary stress to the residents and families [20].

#### 4.4.4. Responses to the Covid-19 Pandemic

Since the outbreak of the COVID-19 pandemic, countries worldwide have tightened their infection control measures to combat the spread of this communicable disease. Residents in the long-term care setting are identified as a high-risk group due to the communal living environment and their compromised immunity levels or health statuses. The Centers for Medicare and Medicaid Services (CMS) had issued a series of regulatory guidelines to all the long-term care facilities including ALFs. This statement was based on an article by BakerTilley on the CMS guidance for long-term care facilities and nursing homes. Accessed 24 October 2021 from: https://www.bakertilly.com/insights/long-term-care-facilities-receive-relief-with-issuance. First, all non-essential visits were banned and most communal dining and group activities were cancelled to reduce the residents’ social contacts in order to prevent virus transmission [28]. Next, ALF staff were required to put on their Personal Protection Equipment (PPE) and screened for COVID-19 symptoms before their duty shifts. While this was highly recommended for ALF staff, many facilities actually faced challenges in acquiring sufficient PPEs for their staff. This statement was derived from Dys, Sarah, Jacyln Winfree, Paula Carder, Sheryle Zimmerman, and Kali S. Thomas. (2021). “Coronavirus Disease 2019 Regulatory Response in United States-Assisted Living Communities: Lessons Learned. Frontiers in Public Health 9:491. https://doi.org/10.3389/fpubh.2021.661042 (accessed 21 October 2021). Some states required staff who tested positive, or displayed COVID-19 symptoms, to remain home. To expand the workforce available in ALFs, CMS also put forth regulatory waivers on training and certification requirements, authorising the “disaster response workers” to provide services without a license until COVID-19 is under control. Under the waiver, nurse aides can postpone their annual training deadlines and feeding assistants can work (under the supervision of nurses) in ALFs with a minimum of one hour of training [29]. Depending on the state’s regulations, some ALFs were also allowed to skip their preadmission screening and transfer residents to another facility without formal discharge. These measures enabled ALF to separate infected residents from those who were tested negative more effectively. Despite all the relief measures, most states have made it mandatory for long-term care facilities to report COVID-19 cases and deaths within a particular duration (daily to weekly). Additional executive orders were also released to increase the frequency of testing for the residents and staff in long-term care settings [28,84]. Finally, states have increased resources by apportioning additional funds to long-term care facilities via different mechanisms to provide disaster relief [28,84].

### 4.5. Micro-Level Regulation

#### 4.5.1. Resident Selection

The ALF market expansion reflected a redistribution of consumers; many of whom would have resided in nursing homes are now in ALFs [77]. To ensure that ALFs can provide adequate care and assistance to suitable residents, most states included facility-specific admission criteria in their legislation. Usually, ALFs were allowed to accept residents with different levels of care needs, including those with higher dependencies [86]. In the US, some admission restrictions were specifically mentioned in the state regulations to allow nursing home admissions only. For instance, a person with 24-h nursing care needs, who is chronically bedridden, has communicable diseases, advanced stage pressure sores/ulcers, or bowel incontinence are encouraged to consider a nursing home or hospital admissions [27,44,72]. Other than that, ALFs also admitted their residents based on their capacities and specialisations. Residents with high physical level of care needs and age-related cognitive declines were more likely to be accepted by authorised facilities, such as the high-frailty ALFs; residents with low physical level of care needs and history of mental illness were more likely to be accepted by behavioural ALFs that possessed the ‘Limited Mental Health License’, such as those in Florida [25].

Pre-admission assessment served as a critical tool to ensure that a resident’s profile fits an ALF’s service scopes. The majority of the states in the US required ALFs to conduct pre-admission assessments for their incoming residents. Even though only 16 states extended such requirements to all licensed settings, 76% of larger ALFs used a standardised tool to screen their residents for potential dementia and cognitive decline [4,29]. Some states also required the clinical history of the applicants, plus various examinations of their mental health states including depression, physical abilities and behavioural patterns before admission [4]. The assessment was generally conducted by a registered nurse who evaluated the compatibility between the individual’s health condition and the existing staff competencies. In order to provide a fair assessment, the nurse must use standardised instruments and techniques to collect information. The American Assisted Living Nursing Association’s assessment practice standards identified a list of domains that should be included during the screening: functionality (ADL), instrumental activities, medication management, safety needs, comprehensive history, lifestyle, perceptions and beliefs, spiritual and cultural beliefs, and social network [49]. While the fit between the facility and resident can be established during the pre-admission assessment, it also enabled ALFs to identify reversible conditions and institute preventive measures before entering the facility [49].

#### 4.5.2. ALF Staff Requirement

Different sets of requirements were proposed in different states to recruit staff in ALF. These requirements vary based on staffing levels, ratios, and staff types [4]. As medical staff, RNs and LPNs were employed based on the state’s requirements for ALFs. Some states allowed RNs to be a facility’s in-charge and provide oversights for multiple facilities, and LPNs/LVNs were required to supervise the care services within an individual facility [7]. However, a medication technician’s work requirement was not well established in the regulatory guidelines [61]. As compared to the other ALF staff, minimal requirements were needed for ALF administrators. With a high school diploma, he/she would need to attend training and continuous education pertaining to the reporting of major incidents and emergency procedures [86].

In the ALFs, a spectrum of medical and care services was offered to the residents. As mentioned in the literature, different training was required to minimise medication errors and reduce hospitalisation rates [58,72]. The training requirements varied in terms of the training hours and topics depending on their job scopes. The general requirements entailed proper licensure/certification, specified training hours, continuing education, and mandatory training on emergency and specialty care. Most states required administrators to obtain their licenses by undergoing state agency-approved training courses. However, specific qualifications or training were not mentioned at the managerial level. The hours for initial training ranged between two to 80 h, while annual training ranged between two to 16 h [77].

Other than the essential restorative services, as well as nursing and personal care training, the most common ALF training curriculums focused on mental health needs, dementia care and emergency responses. Staff were trained to recognise signs of depression and confusion among the residents [44,64,77]. Similar training was also required to manage the needs of residents with cognitive impairment. Many ALFs acknowledged such needs and therefore, required their staff to undergo additional annual continuing education of five to eight hours in dementia care [4,29,77]. Under the domain of emergency response training, several topics were covered, such as training for first aid and cardiopulmonary resuscitation, fire and environmental safety, and infectious disease control [27,44,77]. One study suggested using performance observation as a follow-up measure to evaluate the training outcomes in addition to the standard delivery of the training curriculum. ALFs could utilise the clinical skill checklist (commissioned by the Department of Health, US). The ‘medication administration clinical skills checklist’ is available on the Massachusetts, Department of Health website: https://www.mass.gov/doc/medication-administration-competency-skill-checklist/download (accessed on: 10 June 2021) or the “6 Rights” of Medication Administration “6 Rights” of Medication Administration includes the right patient, medication, dose, time, route and documentation (Mitty and Flores, 2007c) as tools to ensure staff competencies [50] 

**Figure 3:**
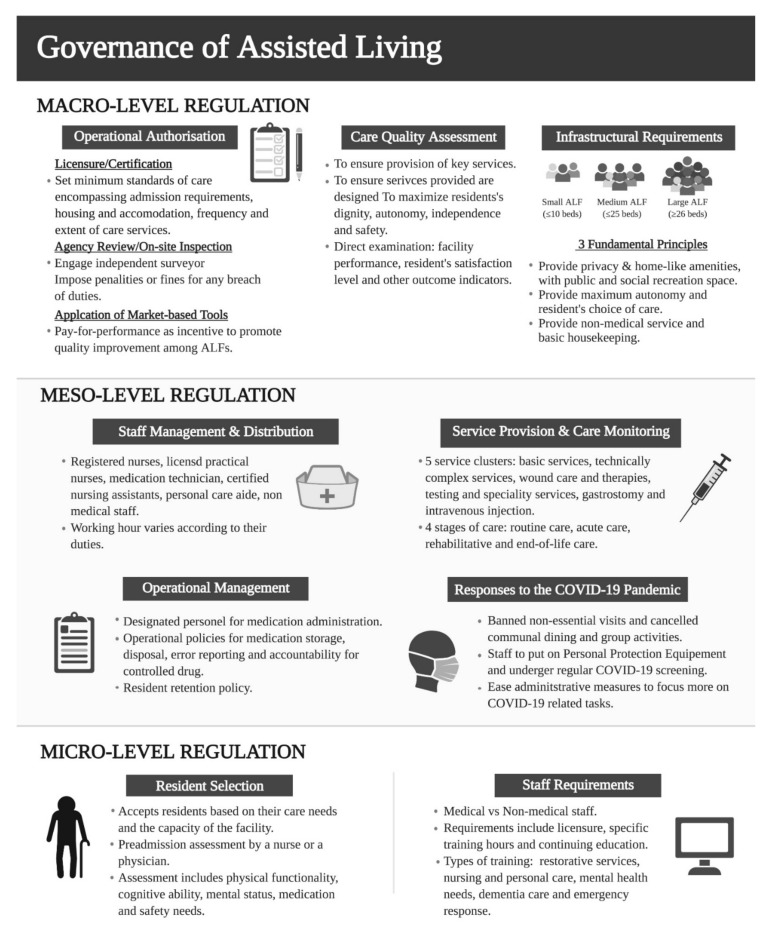
Summary of the macro-, meso- and micro-level regulations of AL consolidated from the review.

Figure 3 illustrates the summary points of the key components of micro-, meso-, and macro-level regulations in the governance of AL. 

## 5. Discussion

AL is an emerging long-term care option that recognises the will and choices of the elderly in maintaining maximum autonomy while receiving care and assistance in a safe and private environment. It serves as an alternative care option for older people instead of the default nursing home option when some of their ADLs are compromised. For individuals with mild to moderate deficiencies in daily living, they may not require the high-intensity care offered in the nursing home. ALFs provide a wider range of health care services that respond to the scheduled and unscheduled needs of the residents [89,94]. A personalised care model, such as the AL model would appeal to individuals with low- to medium-intensity care needs and lessen the care burden of informal caregivers [95]. Therefore, the degree of health care services provided in an ALF generally depends on the need profiles of their residents [43].

This review recommends a set of regulatory guidelines at the micro, meso and macro levels to the AL sectors in many countries on how to best govern the industry while it is still at the budding or maturing stage. These recommendations are not meant to be prescriptive but rather more experimental to enable room for adjustment, especially in jurisdictions whereby AL remains a novel long-term care model. The flexibility given to ALFs to adjust their healthcare services would allow the operators to modulate and optimise their implementation processes to better fit the market demand [20]. In communities with a relatively healthier population, the provider can enter the ALF market as a housing/social model with basic healthcare services available in the facility [8,43,87]. On the other hand, high-frailty ALFs can provide better care for a population with a higher level of physical disabilities [25]. As the desire to age with dignity and a higher degree of autonomy become more prevalent among the elderly, the demand for AL will pave the way to its market expansion, hence diversifying the long-term care options for the older people [22,43].

Even in the US whereby AL is relatively more established conventionally, there has been a lack of standardisation of regulation across the states, and regulatory inaction in certain jurisdictions raised concerns over financial exploitations and care negligence [19,21,23]. Availability of services was affected by the staff and human resources allocation in the facility. Having a mix of RN and LPN was associated with more testing and specialty services; while the presence of licensed nurses increased the availability of basic services [86,87], as well as the total number of services offered [58]. Other than the availability of services, several factors could influence the quality of services provided. For example, the rates of medication error were significantly higher in for-profit ALFs than non-profit ALFs. Similar trends were also observed in ALFs that demanded less training commitment from their staff [55,61,65]. Proper training ensured the quality of service provided and enabled the staff to identify the resident’s needs better. Communication among the ALF staff and external healthcare providers was another critical factor in AL care delivery [68]. Therefore, these factors could serve as potential considerations during the formulation of AL guidelines and regulations.

Nevertheless, there were limitations that ALFs faced in their service deliveries. Not all services needed by the residents can be provided on-site. Sometimes, the residents would need to go to other healthcare facilities to receive diagnostic tests (i.e., mammograms, sigmoidoscopies, stress tests) [94]. ALF staff would also face difficulties in taking care of residents who were reluctant to ask for assistance because they feared that if they requested more help, they might be at risk of being discharged [44]. Similarly, when residents displayed a lack of interest in adhering to the ALF rules or guidelines, they were concerned that this could also put them at risk of not receiving proper care [55,82]. In addition, different residents possessed different sets of needs, with their health statuses constantly evolving, resulting in constant changes of their needs profiles [55]. These scenarios reflected the need to gain a deeper understanding at the individual level and the dynamics between the residents and facility staff.

This review synthesised the implementation of AL through the lens of governance and regulation based on the existing evidence in the US, Canada and Israel. Information from both empirical and conceptual literature were combined and analysed in this review to fill a knowledge gap in the governance and regulation of AL. Consolidation of governance and regulatory experiences of managing AL across these jurisdictions is important to provide a blueprint for other ageing countries that aspire to adopt and adapt AL as a means to diversify the provisions of long-term care.

The major limitation of this review is its limited generalisability, as most of the studies are US-centric despite thorough efforts to gather global evidence. As mentioned above, the local contexts vary from one state to another, therefore, leading to the differences in AL legislations and regulations. Such heterogeneities may also occur in other countries with different long-term care systems and different levels of maturity in service provisions.

The above limitation could shed light on the directions of future studies. For instance, more country-level case studies from different continents, as well as cross-jurisdiction comparative case studies regarding the implementation of AL in different cultures, social institutions and political-economy arrangements are needed. These will facilitate knowledge transfer and policy learning for countries that are grappling to develop the AL market as a way to expand the long-term care options for their ageing populations. Furthermore, perception studies from the older adults regarding the preferences for the arrangement for ALs, including their willingness-to-pay for different services [96,97], and how novel health technologies can be incorporated into their care arrangements, as well as their attitudes and concerns towards the ethical issues, as well as the technological risks that these technologies would bring [98,99,100], would also provide more insights to long-term care policy and practice from the prospective users’ perspectives.

## 6. Conclusions

This review highlighted three levels of regulation—micro-, meso- and macro-levels—that are important in the governance of AL to uphold quality and safeguard the residents’ interests and well-being. The review focused on the core features of AL and its distinction from other conventional models of long-term care, hence supporting the large-scale adoption of AL as a means to diversify long-term care options in ageing countries and societies. The growing number of AL residents reflected that AL is not only popular in North America, but it is also gaining traction among other ageing countries across the world [18].

AL is a care model that encompasses multi-dimensional elements of healthy ageing, which allows its design and service delivery to be more versatile and flexible. While the governments need to drive the effort to ensure proper governance framework and regulatory structure are in place for the implementation of ALFs, the private sectors, including civil society organizations, commercial long-term care providers and the general public, should also contribute to the collective efforts to ensure that the expansion of AL could meet the long-term care needs of the older population without compromising its quality and affordability.

## Data Availability

The summarised data presented in this study are available in the Appendix A and Supplementary Materials. The raw data are available on request from the corresponding author.

## Supplementary Materials

The following are available online at https://www.mdpi.com/article/10.3390/ijerph182111352/s1, Table S1: Critical appraisal of included studies; Table S2: Crowe Critical Appraisal Tool (CCAT) form; Table S3: PRISMA Checklist 2020 [101]; S4: Search Strategies.

## Declaration

The protocol for this review has not been registered.

## Appendix A

**No**	**Study Name/** **Author (s)**	**Country**	**Study Design**	**Study** **Population (s)**	**Types of Long-Term Care Setting**	**Brief Description of the Study**
1	Licensed Nurse Staffing and Health Service Availability in Residential Care and Assisted Living [87]	United States (22 states)	Survey	Licensed nurse	Residential care and assisted living	This study aims to create data-driven typologies of licensed nurse staffing and health services in residential care and assisted living (RC/AL). In this study, a positive correlation between the presence of licensed nurses and the services provided by the RC/AL facilities was observed.
2	Staffing and Service Availability in Assisted Living: The Importance of Nurse Delegation Policies [58]	United States (8 states)	Survey	Facility Staff	Residential care and assisted living	This study identified the possible relationship between nurse delegation practices and the availability of staff and services in RC/AL facilities.
3	State Regulatory Approaches for Dementia Care in Residential Care and Assisted Living [4]	United States (all 50 states)	Qualitative conceptual	Residential care and assisted living facility	Residential care and assisted living	This study investigated the current RC/AL regulations for dementia care. Several important aspects of the regulations, including administrator training, consumer disclosure, physical environment, staffing and pre-admission assessment for dementia were identified.
4	Aging in Place in Assisted Living: Philosophy Versus Policy [59]	United States, Kansas	Survey	Assisted living facility	Assisted living facility	This study examined the RC/AL regulation in Kansas. The authors observed that AL facility policies in Kansas were more restrictive than admission and discharge policies found nationally, especially for regulations focusing on behavioural problems, incontinence, and cognition.
5	Long-Term Care, Residential Facilities, and COVID-19: An Overview of Federal and State Policy Responses [28]	United States	Qualitative conceptual	Various stakeholders in the long-term care systems	Assisted living facility	This study provided an overview of these responses by first summarising federal regulatory changes and then reviewing state-level executive orders.
6	State Medicaid Financing and Access to Large Assisted Living Settings for Medicare–Medicaid Dual-Eligibles [81]	United States	Retrospective cohort study	Adults who live in large (25+ beds) assisted living communities	Assisted living facility	This study aims to provide a basis for understanding the role of Medicaid financing in access to assisted living for duals.
7	Overview of Policies, Guidelines, and Standards for Active Assisted Living (AAL) Data Exchange: Thematic Analysis). [88]	Canada	Survey	Various stakeholders in the long-term care systems	Assisted living facility	This study examined the standards and policy guidelines applicable for the production of AAL technologies. The authors have identified interoperability, privacy and security as the main challenges for data sharing related to AAL technology.
8	Assisted Living Expansion and the Market for Nursing Home Care [2]	United States	Analytical study	Assisted living facility	Assisted living facility and nursing home	This study analysed the effect of market-level changes in assisted living supply on the demand of other long-term care facilities. Their results showed that AL could meet the demand for healthier seniors with more financial resources.
9	Defining Quality in Assisted Living: Comparing Apples, Oranges, and Broccoli [43]	United States	Qualitative conceptual	Various stakeholders in the long-term care systems	Assisted living facility	This study examined the quality of care in AL facilities by focusing on the assessment tools and parameters.
10	Vertical Axes on the Long-Term Care Continuum—A Comparison of Board and Care and Assisted Living [34]	United States	Qualitative conceptual	Various stakeholders in the long-term care systems	Long term care settings	This study examined the effect of public funding on the accessibility of services, availability of resources and the quality of care in the long-term care continuum.
11	Assisted Living and Residential Care in Oregon: Two Decades of State Policy, Supply, and Medicaid Participation Trends [24]	United States, Oregon	Qualitative conceptual	Various stakeholders in the long-term care systems	Long term care settings	This study illustrated the state policies and regulations of Oregon on RC/AL facilities. The authors have identified state financing and reimbursement policies as the key factors driving the market development of apartment-style AL facilities for individuals with financial difficulty.
12	Nuts and Bolts of NP Practice in Assisted Living Facilities [93]	United States	Qualitative conceptual	Nurse practitioner in assisted living facility	Assisted living facility	This study described the practical issues associated with setting up a nurse practitioner (NP) practice in an assisted living facility (ALF), including record keeping, maintaining communication, and scheduling and coordination issues.
13	Variation Across U.S. Assisted Living Facilities: Admissions, Resident Care Needs, and Staffing [75]	United States	Quantitative conceptual	Residential Care Facilities	Residential Care Facilities	This study utilised a nationwide sample of AL facilities in the US to examine different aspects of AL facilities, such as admission policies, resident care needs, and staffing characteristics.
14	Costs, Staffing, and Services of Assisted Living in the United States: A Literature Review [79]	United States	Literature review	Various stakeholders in the long-term care systems	N.A.	This study examined the future trends in ALFs in the United States to determine the impact of health care on costs. The authors have identified trends in the monthly cost of AL facilities, staff turnover and retention rate, and the job vacancy rate.
15	Legal Considerations For Assisted Living Facilities [19]	United States	Qualitative conceptual	Various stakeholders in the long-term care systems	Assisted living facility	This study aims to classify the legal issues facing ALFs. The authors found that regulation and legal regimes concerning ALFs were fragmented, existing almost exclusively on a state-by-state basis.
16	Consumer Satisfaction in Long-Term Care: State Initiatives in Nursing Homes and Assisted Living Facilities [60]	United States	Survey	Long term care residents	Nursing homes and assisted living facilities	This study investigated resident satisfaction in nursing homes and assisted living facilities. The authors observed that this aspect of long-term care services was still under-developed, and easily understandable reports could facilitate better communication between service providers and potential residents.
17	Health Care Services Use in Assisted Living: A Time Series Analysis [26]	Canada, British Columbia	Survey	Assisted living facility residents	Assisted living facility	This study described British Columbia’s regulatory model for assisted living and used time series analysis to examine individuals’ use of health care services before and after moving to assisted living.
18	Assisted living: aging in place and palliative care [44]	United States	Qualitative conceptual	Various stakeholders in the long-term care system	Assisted living facility	This study described the context of assisted living, resident characteristics, key indicators of palliative care, barriers to end-of-life care, and the role, responsibilities, and potential for professional nursing in assisted living.
19	Medication Management in Assisted Living: A National Survey of Policies and Practices [61]	United States	Survey	Assisted living facility	Assisted living facility	This study investigated the medication management of AL facilities. The author found that training for staff responsible for drug administration and assessing residents’ ability to safely self-administer medications were the key factors in reducing medication error.
20	Nursing Delegation and Medication Administration in Assisted Living [7]	Unites States	Qualitative conceptual	Direct care workers in assisted living facilities	Assisted living facility	This study aims to review delegation in AL and to provide recommendations for future practice and research in this area. The authors identified several key variations in the state’s regulation for AL facilities, including personnel providing services, amount and types of service available within the facility.
21	The Taste for Regulation in Long-Term Care [62]	United States	Survey	Long term care specialists	N.A.	This study investigated issues regarding the state’s approach in assuring quality and improving care. Based on the feedback of long-term care specialists, the authors discussed their willingness to stay in the industry and ways to provide better incentives for better care provision.
22	Determinants of the Rigor of State Protection Policies for Persons With Dementia in Assisted Living [85]	United States	Qualitative conceptual	Various stakeholders in the assisted living setting	N.A.	This study examined the adoption of AL protection policies pertaining to staffing, the physical environment, and the use of chemical restraints. The authors found that the rate of state AL protection policy adoptions remained steady over the study period, with staffing policies becoming less rigorous over time.
23	Mistreatment in Assisted Living Facilities: Complaints, Substantiations, and Risk Factors [21] Si	United Status	Survey	Assisted living facilities	Assisted living facility	This study investigated the potential factors that may contribute to the complaints and mistreatments in AL facilities. The authors found that the size and ownership of the facility and the availability of personal care services influenced the rate of complaints and allegations.
24	Organizational Determinants of Resident Satisfaction With Assisted Living [63]	Unites States, Maryland	Survey	Residents in assisted living facilities	Assisted living facility	This study investigated the potential factors that may influence resident satisfaction in AL facilities. The authors found that the size and ownership of the facility, availability of physical amenities, and personal space often determined residents’ satisfaction levels.
25	Increasing Prevalence of Assisted Living as a Substitute for Private—Pay Long—Term Nursing Care [76]	United States	Quantitative conceptual	Nursing homes and assisted living facilities	Nursing homes and assisted living facilities	This study estimated the effect of a change in county-level assisted living beds on the prevalence of private-pay residents and private-pay resident days at the nursing home level. The authors suggested that enhancing AL facility capacity influenced the resident intake and lengths of stay for the nursing home.
26	Connecting policy to licensed assisted living communities, introducing health services regulatory analysis ([29]	United States	Qualitative conceptual	Various stakeholders in the assisted living setting	Assisted living facilities with dementia care unit	This study described the state AL policies and regulations for dementia care. The authors observed that the adoptions of policies were only limited to dementia—designated settings but not general AL facilities with demented residents.
27	The Place of Assisted Living in Long-Term Care and Related Service Systems [8]	United States	Qualitative conceptual	Various stakeholders in the long-term care system	N.A.	This study discussed the role of AL in the long-term care continuum. The authors examined the different models of AL and the relevant federal and state policy rules.
28	The Effect of Licensure Type on the Policies, Practices, and Resident Composition of Florida Assisted Living Facilities [25]	United States, Florida	Survey	Various stakeholders in the assisted living setting	Assisted living facility	This study characterised the AL facilities in Florida based on their licensure profiles. In addition, the authors have identified the characteristics of residents and admission-discharge policies in relation to the facility’s licensure profiles.
29	Variability and Potential Determinants of Assisted Living State Regulatory Stringency [72]	United States	Observational study	Various stakeholders in the assisted living setting	N.A.	This study examined state variations in assisted living (AL) regulatory policies for admission/retention, staffing/training, medication management, and dementia care. Factors associated with domain-specific and overall regulatory stringency were identified.
30	State Variability in the Prevalence and Healthcare Utilization of Assisted Living Residents with Dementia [73]	United States	Observational national study	LTC residents with ADRD	Assisted living facility, nursing homes and the community	This study described the state variability in the prevalence of Alzheimer’s disease and related dementias (ADRD) among Medicare beneficiaries residing in larger (25+ bed) ALs and their healthcare utilization. The authors observed that the status of ADRD influenced the choice of long-term care facility by the resident and the rate of hospitalization in the respective facilities.
31	Comparing residential long-term care regulations between nursing homes and assisted living facilities [77]	United Status	Analytical study	Various stakeholders in the long-term care system	N.A.	This study examined the governance of AL facilities in the entire US and compares it to the regulation of nursing homes (NH). The authors observed that the state ALF regulations were less stringent than NH in all categories.
32	Assisted Living Facilities as a Site for NP Practice [86]	United States	Literature review	Various stakeholders in the assisted living setting	N.A.	This study illustrated the characteristics of AL facilities in the US. The authors covered different aspects of the AL facility, including the nature of the setting, AL philosophy, types of facility infrastructure and profile of residents.
33	We’ve got trouble: medications in assisted living [64]	United States	Survey	Assisted living facilities	N.A.	This study examined the factors that influence medication-related problems in AL facilities. The authors conducted surveys for 1335 AL facilities and focused on the relationship between the allegations and the nature of the issues, and facility characteristics.
34	Definition and Classification of Assisted Living [78]	United States	Literature review	Various stakeholders in the assisted living setting	N.A.	This review discussed the existing AL typologies and addresses matters related to the AL facility’s structure, process, population, and philosophy to varying degrees.
35	Medication Administration Errors in Assisted Living: Scope, Characteristics, and the Importance of Staff Training [65]	United States	Comparative study (by observation and survey)	All staff who prepared or passed medications in the assisted living facility	Assisted living facility	This study investigated the correlation between training received by staff and medication error in AL facilities. The authors observed that non-nurses committed more errors than medication aides and licensed practical nurses.
36	Market Structure, Competition from Assisted Living Facilities, and Quality in the Nursing Home Industry [66]	United States, Ohio	Survey	Various stakeholders in the long-term care system	Nursing home and assisted living facility	This paper examined data from the State of Ohio to determine if the nursing home market structure and the expansion in the supply of assisted living beds impacted the quality of care provided by nursing homes.
37	Variability in State Regulations Pertaining to Infection Control and Pandemic Response in US Assisted Living Communities [84]	United States	Qualitative systematic analysis	Various stakeholders in the long-term care system	Assisted living facility	This study examined the role of RC/AL communities in preparing and protecting their residents during a disease outbreak.
38	Rooms Without Rules: Shaping Policies for Assisted Living Facilities [20]	United States	Qualitative conceptual	Various stakeholders in the assisted living setting	N.A.	This article described the issues surrounding policy making for assisted living with an eye toward promoting the role of nurses in this important policy area.
39	Community Care Alternatives for Older Adults [35]	United States	Qualitative conceptual	Various stakeholders in the long-term care system	N.A.	This paper illustrated the concept of AL and the practical aspects of the AL facility, including service provided and infrastructural design. By comparing the different AL models, the authors concluded that it is more important for the service providers to identify models that suit their communities and residents.
40	Assisted-living for older people in Israel: market control or government regulation? [22]	Israel	Qualitative conceptual	Various stakeholders in the assisted living setting in Israel	N.A.	This review provided a balanced discussion regarding the importance of formal legal regulation in protecting the interest of frail AL residents in Israel. The authors concluded that formal direct regulation was not the best route to follow but that the better course would be to develop new ‘combined’ regulatory legislation.
41	State Financing Programs for Long-Term Care Facilities [67]	United States	Survey	Various stakeholders in the long-term care system	N.A.	This study examined the size and scope of loan programs for long-term care by surveying various state loan agencies. Though there were variations in financing across states, the state programs were important sources of long-term care financing.
42	The Coming Home Program: Creating a State Road Map for Affordable Assisted Living Policy, Programs, and Demonstrations [45]	Unites States	Qualitative conceptual	Various stakeholders in the assisted living setting	Assisted living projects	This paper described barriers and opportunities to creating affordable assisted living facilities for older persons eligible for Medicaid services. The paper also explained the need for affordable assisted living, Coming Home’s definition of affordable assisted living, and the structure of the Coming Home Program.
43	Improving Practice Through Research In and About Assisted Living: Implications for a Research Agenda [46]	United States	Qualitative conceptual	Various stakeholders in the assisted living setting	N.A.	This paper explained the concept of AL, including the past understanding and the future directions for research.
44	Policies to Protect Persons With Dementia in Assisted Living: Déjà Vu All Over Again? [23]	United States	Qualitative conceptual	Various stakeholders in the assisted living setting	N.A.	This study examined the states’ regulatory practices and the underlying rationales for AL residents with dementia. The authors focused on domains closely related to the safety and protection of persons with dementia, including environmental features, staffing, and the use of chemical restraints.
45	Assisted Living: Shall We Learn from History or Repeat it? [47]	United States	Qualitative conceptual	Various stakeholders in the assisted living setting	N.A.	This study reported the demographic and profile of AL residents. In addition, the author observed a spectrum of medical illnesses and medication needs across all the AL residents.
46	Assisted Living Nursing Practice: Medication Management: Part 1 Assessing the Resident for Self-Medication Ability [48]	United States	Qualitative conceptual	Nurses in assisted living	Assisted living facility	This article discussed assessment criteria of self-medication ability, drawn from a variety of instruments. In keeping with assisted living nursing standards of practice, the assisted living nurse has a critical responsibility in the assessment of this self-care ability.
47	Assisted Living Nursing Practice: Medication Management: Part 2 Supervision and Monitoring of Medication Administration by Unlicensed Assistive Personnel [50]	United States	Qualitative conceptual	Nurses in assisted living	Assisted living facility	This article addressed delegation, standards of practice of medication administration, types of medication errors, the components of a performance evaluation tool, and a culture of safety.
48	Assisted Living Nursing Practice: Admission Assessment [49]	United States	Qualitative conceptual	Nurses in assisted living	Assisted living facility	This article examined the existing standards of practice as recommended by the American Assisted Living Nurses Association. The role of the Licensed Practical Nurse/Licensed Vocational Nurse in resident assessment was also discussed.
49	Trends in State Regulation of Assisted Living [51]	United States	Qualitative conceptual	Various stakeholders in the assisted living setting	N.A.	The author assessed regulatory practices for assisted living across the country and provided a summary perspective based on his extensive work on this issue over the past decade.
50	Assisted living: a regulation dilemma. Improving assisted living is no easy job, lawmakers have to look at quality, economy and affordability [52]	United States	Qualitative conceptual	Various stakeholders in the assisted living setting	N.A.	This review discussed a few essential aspects of AL: market evolution, regulation in quality assurance, and cost and financing. The author also highlighted potential challenges, including keeping AL affordable and expanding the AL market to provide more options for would-be residents.
51	What Will Long-Term Care Be Like in 2040? [53]	United States	Qualitative conceptual	Various stakeholders in the long-term care system	N.A.	This article discussed the key forces that will shape the future include the aging of the baby-boomer generation, personal choice, concerns about quality, new technologies, dementia research, payment issues, financial pressures, and workforce needs.
52	Sizing Up The Market For Assisted Living [74]	United States	Quantitative conceptual	Various stakeholders in the assisted living setting	N.A.	This study is a country-level assessment of the AL supply market. The authors observed that more AL facilities were located in areas with higher educational attainment, income, and housing wealth.
53	Quality Concerns in Assisted Living Facilities [54]	United States	Qualitative conceptual	Various stakeholders in the assisted living setting	N.A.	This paper examined the various challenges in assisted living settings. The authors highlighted the lack of national regulatory standards as the key issue in the quality assurance of long-term care in AL facilities.
54	Understanding the Intersection of Individual Needs and Choices With Organizational Practices: The Case of Medication Management in Assisted Living [82]	United States, Maryland	Ethnographic study	Six assisted living facilities in Maryland	Assisted living facility	This study discussed the need to balance residents’ needs and choices for medication administration. While service providers upheld their setting-specific practices, there might be gaps between the service offered and residents’ needs and preferences.
55	Comparing Needed Training With Topics of Interest Among Assisted-Living Providers [55]	United States, Washington State	Qualitative conceptual	Various stakeholders in the assisted living setting	N.A.	This article described a new quality improvement program developed for assisted-living facilities in Washington State and compare needed training with topics of interest of care providers in these assisted-living facilities.
56	Individualization and the Health Care Mosaic in Assisted Living [69]	United States, Georgia	Survey	Assisted living residents and their care network members	Assisted Living Facility	This study investigated the AL consumers’ experiences by surveying the residents and their care network members. The authors observed that AL service providers offered different types of health care based on the needs of their residents. This approach allowed the facility to develop individualised health care for its residents.
57	Communicative Competence: Responding to Residents’ Health Changes in Assisted Living [68]	United States	Survey	Residents and their care convoy members from eight diverse AL communities	Assisted living communities	This study examined the care convoy communication during times of residents’ health changes in AL. The authors observed that the ability of the service providers to effectively communicate with their residents during the change was influenced by factors, such as the change and resident, informal and formal caregiver, convoy, AL community, and regulatory influences.
58	Admission and Continued—Stay Criteria for Assisted Living Facilities [6]	United States	Qualitative conceptual	Various stakeholders in the assisted living setting	N.A.	This paper proposed a set of admission and continued stay criteria for individuals residing in assisted living that could serve as a guideline for state regulations in addressing the balance between safety and autonomy in ALFs is recommended.
59	Infection Prevention and Control Standards in Assisted Living Facilities: Are Residents’ Needs Being Met? [27]	United States	Qualitative conceptual	Various stakeholders in the assisted living setting	N.A.	This paper examined the legal aspects of the AL facility, including living admissions criteria, medical oversight, medication administration, vaccination requirements, and standards for infection control training. The authors observed a lack of standard regulation for the AL market in the US.
60	Managed Care and Assisted Living: Trends and Future Prospects [56]	United States	Qualitative conceptual	Various stakeholders in the assisted living setting	N.A.	This paper examined the move toward managed care and the increasing interest in integrating acute and long-term care for dual eligibles, which signaled a growing role for assisted living facilities as a major resource for elders who needed a supportive and service-rich residential living environment.
61	Infection Control Practices in Assisted Living Facilities: A Response to Hepatitis B Virus Infection Outbreaks [70]	United States, Virginia	Survey	Assisted living facilities in central Virginia	Assisted living facility	This study examined the policies and regulations of AL facilities in response to disease outbreaks in Virginia. The authors observed that some facilities did not adhere to the recommendations stated in federal guidelines.
62	Cohort differences in dementia recognition and treatment indicators among assisted living residents in Maryland: did a change in the resident assessment tool make a difference? [71]	United States, Maryland	Survey	Demented residents in assisted living facilities	Assisted living facility	This study examined cohort differences in dementia recognition and treatment indicators between two cohorts of AL residents with dementia and evaluated the changes prior to and following a dementia-related policy modification to more adequately assess memory and behavioral problems.
63	Physician Perspectives on Medical Care Delivery in Assisted Living [83]	United States	Cross-sectional descriptive study	Physicians and administrators of 125 AL settings in which they had patients.	Assisted living facility	This study examined the medical care provided by physicians in AL facilities. The authors observed challenges for AL staff to provide medical services that required more professional skills and knowledge.
64	Variation in Hospice Services by Location of Care: Nursing Home Versus Assisted Living Facility Versus Home [80]	United States	Retrospective cohort study	Hospice patients who received routine hospice care	Hospice care settings	This study described differences in hospice services for patients living at home, in nursing homes, or in assisted living facilities, including the overall number and duration of visits by different hospice care providers across varying lengths of stay. The authors observed significant differences between characteristics of hospice patients in different settings, as well as the mix of services they received.
65	Coalition to change assisted living regulations governing special units for dementia patients [57]	N.A.	Qualitative conceptual	Various stakeholders in the assisted living setting	N.A.	This report highlighted the difficulties and challenges faced by ALF with special dementia care unit.
