# Supplementary material for: Governance of Assisted Living in Long-Term Care: A Systematic Literature Review
Source: Int J Environ Res Public Health. 2021 Oct 28;18(21):11352. doi: 10.3390/ijerph182111352 (PMC8583175; doi:10.3390/ijerph182111352)
Supplement: Supplementary file 1 [file ijerph-18-11352-s001.zip › ijerph-1413554-supplementary.pdf]

Supplementary Data

Table S1. Critical appraisal of included studies.

	**Scores**															
**Category items**	Beeber et al., 2014	Beeber et al., 2018	Carder, 2017	Chapin and Dobbs-Kepper, 2001	Chen et al., 2020	Fabius et al., 2021	Fadrique et al., 2020	Grabowski et al., 2012	Hawes and Phillips, 2007	Herd, 2001	Hernandez, 2007	Holcomb and Wink, 2002	Han et al., 2016	Kisling-Rundgren et al., 2016	Yang, 2015	Lowe et al., 2003
1) Preliminaries	4	5	4	5	4	4	5	4	4	4	5	4	5	5	5	5
2) Introduction	5	5	5	4	5	5	5	5	4	4	5	5	5	5	5	5
3) Design	4	4	3	4	4	3	5	5	3	3	4	2	4	5	0	4
4) Sampling	4	4	4	4	0	5	4	5	0	3	4	0	5	4	0	4
5) Data Collection	3	4	5	4	3	3	4	4	0	3	4	0	4	4	0	5
6) Ethical matters (participant ethics and researcher ethics)	5	5	4	2	5	5	5	4	5	2	3	0	4	4	0	5
7) Results	5	5	4	4	4	4	5	5	5	4	5	4	4	5	5	5
8) Discussion	4	4	5	5	5	5	4	5	4	4	4	3	5	5	4	5
9) Aggregate Scores (/40)	34	36	34	32	30	34	37	37	25	27	34	18	36	37	19	38

**Category items**	McGrail et al., 2013	Mitty, 2004	Mitty, 2009	Mitty et al., 2010	Mor and Alan, 2010	Nattinger and Kaskie, 2017	Phillips and Guo, 2011	Sikorska, 1999	Silver et al., 2018	Smith et al., 2021	Stone and Reinhard, 2007	Street et al., 2009	Temkin-Greener et al., 2020	Thomas et al., 2020	Trinkoff et al., 2019	Wink and Holcomb, 2002	Woods et al., 2010
1) Preliminaries	5	5	5	5	5	5	5	5	5	5	4	5	5	5	4	4	5
2) Introduction	5	5	5	5	5	5	5	5	5	5	5	5	5	4	5	5	5
3) Design	5	4	4	0	5	5	4	5	5	5	3	4	4	4	4	3	5
4) Sampling	5	4	5	0	4	5	5	4	5	4	0	4	4	5	5	0	4
5) Data Collection	4	3	4	0	4	4	4	5	4	4	0	3	4	5	4	0	4
6) Ethical matters (participant ethics and researcher ethics)	5	3	4	4	5	0	5	3	3	5	0	5	5	4	4	0	2
7) Results	5	4	5	5	5	5	4	5	4	5	4	4	4	4	4	4	5
8) Discussion	4	4	5	5	4	4	5	5	5	5	4	5	5	4	4	3	4
9) Aggregate Scores (/40)	38	32	37	24	37	34	37	37	36	38	20	35	36	35	34	19	34

Table S1. Critical appraisal of included studies *cont*.

Category items	Scores																
	Zimmerman and Sloane, 2007	Zimmerman et al., 2011	Bowblis, 2012	Bucy et al., 2020	Chen and Cohen, 2002	Denham, 2018	Doron and Lightman, 2003	Harrington et al., 2002	Jekens et al., 2005	Kane and Wilson, 2007	Kaskie et al., 2015	Levenson, 2008	Mitty and Flores, 2007a	Mitty and Flores, 2007b	Mitty and Flores, 2007c	Mollica, 2008	Retsinas, 2005
1) Preliminaries	4	5	5	4	4	3	4	5	4	3	4	0	3	4	4	0	0
2) Introduction	5	5	3	3	4	5	5	5	5	5	5	5	5	4	4	5	3
3) Design	4	5	5	3	0	0	0	4	4	0	4	0	3	4	3	0	0
4) Sampling	0	4	5	3	0	0	0	5	4	0	0	0	0	0	0	0	0
5) Data Collection	0	5	4	3	0	0	0	4	5	0	0	0	0	0	0	0	0
6) Ethical matters (participant ethics and researcher ethics)	4	4	4	5	0	0	0	4	3	0	4	0	0	0	0	0	0
7) Results	4	5	4	4	4	4	5	5	4	2	5	5	4	4	4	5	5
8) Discussion	4	5	5	3	3	4	4	3	5	4	4	3	4	4	4	3	3
9) Aggregate Scores (/40)	25	38	35	28	15	16	18	35	33	14	26	13	19	20	19	13	11

Category items	Sloane et al., 2014	Stevenson and Grabowski, 2010	Aud and Rantz, 2004	Carder et al., 2009	Dupler and Crogan, 2004	Kemp et al., 2019a	Kemp et al., 2019b	Kissam et al., 2003	Kossover et al., 2014	Mollica, 1998	Patel et al., 2009	Samus et al., 2013	Sloanne et al., 2011	Unroe et al., 2017	Yeaworth, 2000
1) Preliminaries	4	5	0	5	5	4	4	4	5	4	5	5	5	5	0
2) Introduction	5	5	3	5	5	5	5	3	5	5	5	5	5	5	5
3) Design	0	5	0	4	4	4	4	3	4	4	4	5	4	4	0
4) Sampling	0	5	0	3	5	4	4	0	4	5	4	5	5	4	0
5) Data Collection	0	4	0	5	5	5	5	0	4	4	5	5	4	4	0
6) Ethical matters (participant ethics and researcher ethics)	4	4	0	4	0	5	5	3	3	2	3	4	4	4	0
7) Results	4	5	5	4	4	4	5	3	5	4	5	4	4	4	5
8) Discussion	4	5	3	5	4	5	5	4	4	4	5	4	4	4	0
9) Aggregate Scores (/40)	21	38	11	35	32	36	37	20	34	32	36	37	36	34	10

Table S2. Crowe Critical Appraisal Tool (CCAT) form

Category item	Item descriptors	Description	Score (1-5)
	**1. Preliminaries**		
Title	1. Includes study aims and designs		
Abstract	1. Key information<br>2. Balanced and informative		
Last	1. Sufficient detail others could reproduce<br>2. Clear/concise writing, table(s), diagram(s) and figure(s)		
	**Preliminaries (/5)**		
	**2. Introduction**		
Background	1. Summary of current knowledge<br>2. Specific problem(s) addressed and reason(s) for addressing		
Objective	1. Primary objective(s), hypothes(es), or aim(s)<br>2. Secondary question(s)		
	**Is it worth continuing?**	**Introduction (/5)**	
	**3. Design**		
Research design	1. Research design chosen and why<br>2. Suitability of research design(s)		
Intervention, treatment, exposure	1. Intervention(s)/ treatment(s)/ exposure(s) chosen and why<br>2. Precise details of intervention(s)/ treatment(s)/ exposure(s) for each group<br>3. Intervention(s)/ treatment(s)/ exposure(s) valid and reliable		
Outcome, output, predictor, measure	1. Outcome(s)/ output(s)/ predictor(s)/ measure(s) chosen and why<br>2. Clearly define outcome(s)/ output(s)/ predictor(s)/ measure(s)<br>3. Outcome(s)/ output(s)/ predictor(s)/ measure(s) valid and reliable		
Bias, etc	1. Potential bias, confounding variables, effect modifiers, interactions<br>2. Sequence generation, group allocation, group balance, and by whom<br>3. Equivalent treatment of participants/ cases/ groups		
	**Is it worth continuing?**	**Design (/5)**	
	**4. Sampling**		
Sampling method	1. Sampling method(s) chosen and why<br>2. Suitability of sampling method		
Sampling size	1. Sampling size, how chosen and why<br>2. Suitability of sample size		
Sampling protocol	1. Target/actual/sample population(s): description and suitability<br>2. Participants/cases/groups: inclusion and exclusion criteria<br>3. Recruitment of participants/cases/groups		
	**Is it worth continuing?**	**Sampling (/5)**	
	**5. Data collection**		
Collection method	1. Collection method(s) chosen and why<br>2. Suitability of collection method(s)		
Collection protocol	1. Include date(s), location(s), setting(s), personnel, material(s), process(es)<br>2. Methods to ensure/ enhance quality of measurement/ instrumentation<br>3. Manage non-participation, withdrawal, incomplete/ lost data		
	**Is it worth continuing?**	**Data collection (/5)**	
	**6. Ethical matters**		
Participant ethics	1. Informed consent, equity<br>2. Privacy, confidentiality/ anonymity		
Researcher ethics	1. Ethical approval, funding, conflict(s) of interest<br>2. Subjectivities, relationship(s) with participants/ cases		
	**Is it worth continuing?**	**Ethical matters (/5)**	
	**7. Results**		
Analysis, Integration, Interpretation method	1. A.I.I. method(s) for primary outcome(s)/ output(s)/ predictor(s) chosen and why<br>2. Additional A.I.I. methods (e.g. subgroup analysis) chosen and why<br>3. Suitability of analysis/ integration/ interpretation method		
Essential analysis	1. Flow of participants/ cases/ groups through each stage of research<br>2. Demographic and other characteristics of participants/ cases/ groups<br>3. Analyse raw data, response rate, non-participation/ withdrawal/ incomplete/lost data		
Outcome, output, predictor analysis	1. Summary of results and precision for each outcome/ output/ predictor/ measure<br>2. Consideration of benefits/ harms, unexpected results, problems/ failures		

	3. Description of outlying data (e.g. diverse cases, adverse effects, minor themes)	
		**Results (/5)**
	**8. Discussion**	
Interpretation	1. Interpretation of results in the context of current evidence and objectives<br>2. Draw inferences consistent with the strength of data<br>3. Consideration of alternative explanations for observed results<br>4. Account for bias, confounding/ effect modifiers/ interactions/ imprecision	
Generalisation	1. Consideration of overall practical usefulness of the study<br>2. Description of generalisability (external validity) of the study	
Concluding remarks	1. Highlight study's particular strength<br>2. Suggest steps that may improve future results (e.g. limitations)<br>3. Suggest further studies	
		**Discussion (/5)**
	**9. Total**	
Total score	1. Add all scores for categories 1-8	
		**Total (/40)**

Note: Scoring for each category is based on the guiding principles recommended in the Crowe Critical Appraisal Tool (CCAT) User Guide Version 1.4 (Crowe 2013)

Table S3. PRISMA Checklist 2020

Section and Topic	Item #	Checklist item	Location where item is reported
**TITLE**			
Title	1	Identify the report as a systematic review.	Page 1
**ABSTRACT**			
Abstract	2	See the PRISMA 2020 for Abstracts checklist.	Page 1
**INTRODUCTION**			
Rationale	3	Describe the rationale for the review in the context of existing knowledge.	Section 1.4 Page 2-3
Objectives	4	Provide an explicit statement of the objective(s) or question(s) the review addresses.	Section 1.4 Page 2-3
**METHODS**			
Eligibility criteria	5	Specify the inclusion and exclusion criteria for the review and how studies were grouped for the syntheses.	Section 3.2 Page 4-5
Information sources	6	Specify all databases, registers, websites, organisations, reference lists and other sources searched or consulted to identify studies. Specify the date when each source was last searched or consulted.	Section 3.1 Page 4
Search strategy	7	Present the full search strategies for all databases, registers and websites, including any filters and limits used.	Section 3.1 Page 4
Selection process	8	Specify the methods used to decide whether a study met the inclusion criteria of the review, including how many reviewers screened each record and each report retrieved, whether they worked independently, and if applicable, details of automation tools used in the process.	Section 3.1 Page 4
Data collection process	9	Specify the methods used to collect data from reports, including how many reviewers collected data from each report, whether they worked independently, any processes for obtaining or confirming data from study investigators, and if applicable, details of automation tools used in the process.	Section 3.1 Page 4
Data items	10a	List and define all outcomes for which data were sought. Specify whether all results that were compatible with each outcome domain in each study were sought (e.g. for all measures, time points, analyses), and if not, the methods used to decide which results to collect.	NA
	10b	List and define all other variables for which data were sought (e.g. participant and intervention characteristics, funding sources). Describe any assumptions made about any missing or unclear information.	NA
Study risk of bias assessment	11	Specify the methods used to assess risk of bias in the included studies, including details of the tool(s) used, how many reviewers assessed each study and whether they worked independently, and if applicable, details of automation tools used in the process.	Section 3.4 Page 5
Effect measures	12	Specify for each outcome the effect measure(s) (e.g. risk ratio, mean difference) used in the synthesis or presentation of results.	NA
Synthesis methods	13a	Describe the processes used to decide which studies were eligible for each synthesis (e.g. tabulating the study intervention characteristics and comparing against the planned groups for each synthesis (item #5)).	Section 3.5 Page 5
	13b	Describe any methods required to prepare the data for presentation or synthesis, such as handling of missing summary statistics, or data conversions.	Section 3.5 Page 5
	13c	Describe any methods used to tabulate or visually display results of individual studies and syntheses.	Section 3.5 Page 5
	13d	Describe any methods used to synthesize results and provide a rationale for the choice(s). If meta-analysis was performed, describe the model(s), method(s) to identify the presence and extent of statistical heterogeneity, and software package(s) used.	Section 3.5 Page 5

Section and Topic	Item #	Checklist item	Location where item is reported
	13e	Describe any methods used to explore possible causes of heterogeneity among study results (e.g. subgroup analysis, meta-regression).	NA
	13f	Describe any sensitivity analyses conducted to assess robustness of the synthesized results.	NA
Reporting bias assessment	14	Describe any methods used to assess risk of bias due to missing results in a synthesis (arising from reporting biases).	NA
Certainty assessment	15	Describe any methods used to assess certainty (or confidence) in the body of evidence for an outcome.	NA
**RESULTS**			
Study selection	16a	Describe the results of the search and selection process, from the number of records identified in the search to the number of studies included in the review, ideally using a flow diagram.	Section 4.1 Page 6
	16b	Cite studies that might appear to meet the inclusion criteria, but which were excluded, and explain why they were excluded.	NA
Study characteristics	17	Cite each included study and present its characteristics.	Section 4.1 Page 6
Risk of bias in studies	18	Present assessments of risk of bias for each included study.	Section 4.2 Page 7
Results of individual studies	19	For all outcomes, present, for each study: (a) summary statistics for each group (where appropriate) and (b) an effect estimate and its precision (e.g. confidence/credible interval), ideally using structured tables or plots.	Appendix
Results of syntheses	20a	For each synthesis, briefly summarise the characteristics and risk of bias among contributing studies.	NA
	20b	Present results of all statistical syntheses conducted. If meta-analysis was done, present for each the summary estimate and its precision (e.g. confidence/credible interval) and measures of statistical heterogeneity. If comparing groups, describe the direction of the effect.	NA
	20c	Present results of all investigations of possible causes of heterogeneity among study results.	NA
	20d	Present results of all sensitivity analyses conducted to assess the robustness of the synthesized results.	NA
Reporting biases	21	Present assessments of risk of bias due to missing results (arising from reporting biases) for each synthesis assessed.	NA
Certainty of evidence	22	Present assessments of certainty (or confidence) in the body of evidence for each outcome assessed.	NA
**DISCUSSION**			
Discussion	23a	Provide a general interpretation of the results in the context of other evidence.	Section 5 Page 15
	23b	Discuss any limitations of the evidence included in the review.	Section 5 Page 16
	23c	Discuss any limitations of the review processes used.	Section 5 Page 16
	23d	Discuss implications of the results for practice, policy, and future research.	Section 5 Page 16
**OTHER INFORMATION**			
Registration and protocol	24a	Provide registration information for the review, including register name and registration number, or state that the review was not registered.	NA
	24b	Indicate where the review protocol can be accessed, or state that a protocol was not prepared.	NA
	24c	Describe and explain any amendments to information provided at registration or in the protocol.	NA
Support	25	Describe sources of financial or non-financial support for the review, and the role of the funders or sponsors in the review.	Page 17
Competing interests	26	Declare any competing interests of review authors.	Page 17

Section and Topic	Item #	Checklist item	Location where item is reported
Availability of data, code and other materials	27	Report which of the following are publicly available and where they can be found: template data collection forms; data extracted from included studies; data used for all analyses; analytic code; any other materials used in the review.	Page 17

101. Page, M.J.; McKenzie, J.E.; Bossuyt, P.M.; Boutron, I.; Hoffmann, T.C.; Mulrow, C.D.; Shamseer, L.; Tetzlaff, J.F.; Akl, E.A.; Brennan, S.E; et al. The PRISMA 2020 statement: an updated guideline for reporting systematic reviews *BMJ* **2021**; *372:n71*, doi:10.1136/bmj.n71.

S4. Search Strategies

Scopus Search Strategy:
(TITLE-ABS-KEY ("assisted living" OR "assisted-living") AND TITLE-ABS-KEY ("financ*" OR "governance" OR "regulat*" OR "law" OR "legislat*"))

PubMed Search Strategy:

("assisted living" OR "assisted-living") AND ("financ*" OR "governance" OR "regulat*" OR "law" OR "legislat*")

Medline Search Strategy:

("assisted living" OR "assisted-living") AND ("financ*" OR "governance" OR "regulat*" OR "law" OR "legislat*")
